# Supplementary figures and images for: OX2R-selective orexin agonism is sufficient to ameliorate cataplexy and sleep/wake fragmentation without inducing drug-seeking behavior in mouse model of narcolepsy
Source: PLoS One. 2022 Jul 22;17(7):e0271901. doi: 10.1371/journal.pone.0271901 (PMC9307173; doi:10.1371/journal.pone.0271901)

Supplemental Figure 1

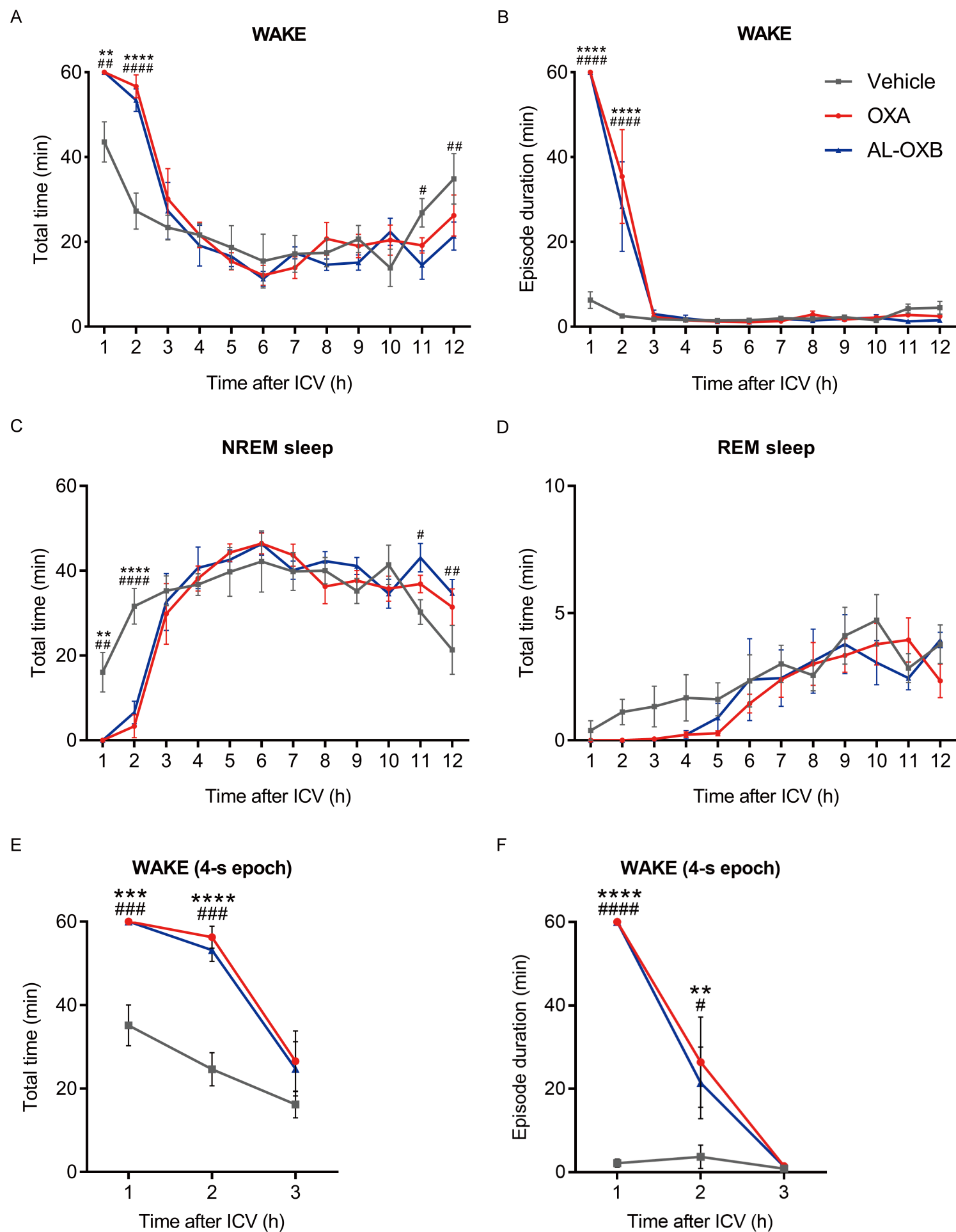

Supplement: S1 Fig — (A-D) Hourly plots of wake time (A), wake episode duration (B), NREM sleep time (C) and REM sleep time (D) during dark phase after ICV administration. (E and F) First 3 hours of the same EEG/EMG data analyzed in 4-s epochs; wake time (E) and wake episode duration (F). Data represent the means ± SEM from 6 mice. *p < 0.05, **p < 0.01, ****p < 0.0001 for OXA vs. vehicle; #p < 0.05, ##p < 0.01, ####p < 0.0001 for AL-OXB vs. vehicle; two-way repeated-measures ANOVA followed by Bonferroni’s multiple comparisons test. (PDF) [file pone.0271901.s001.pdf]

Supplemental Figure 2

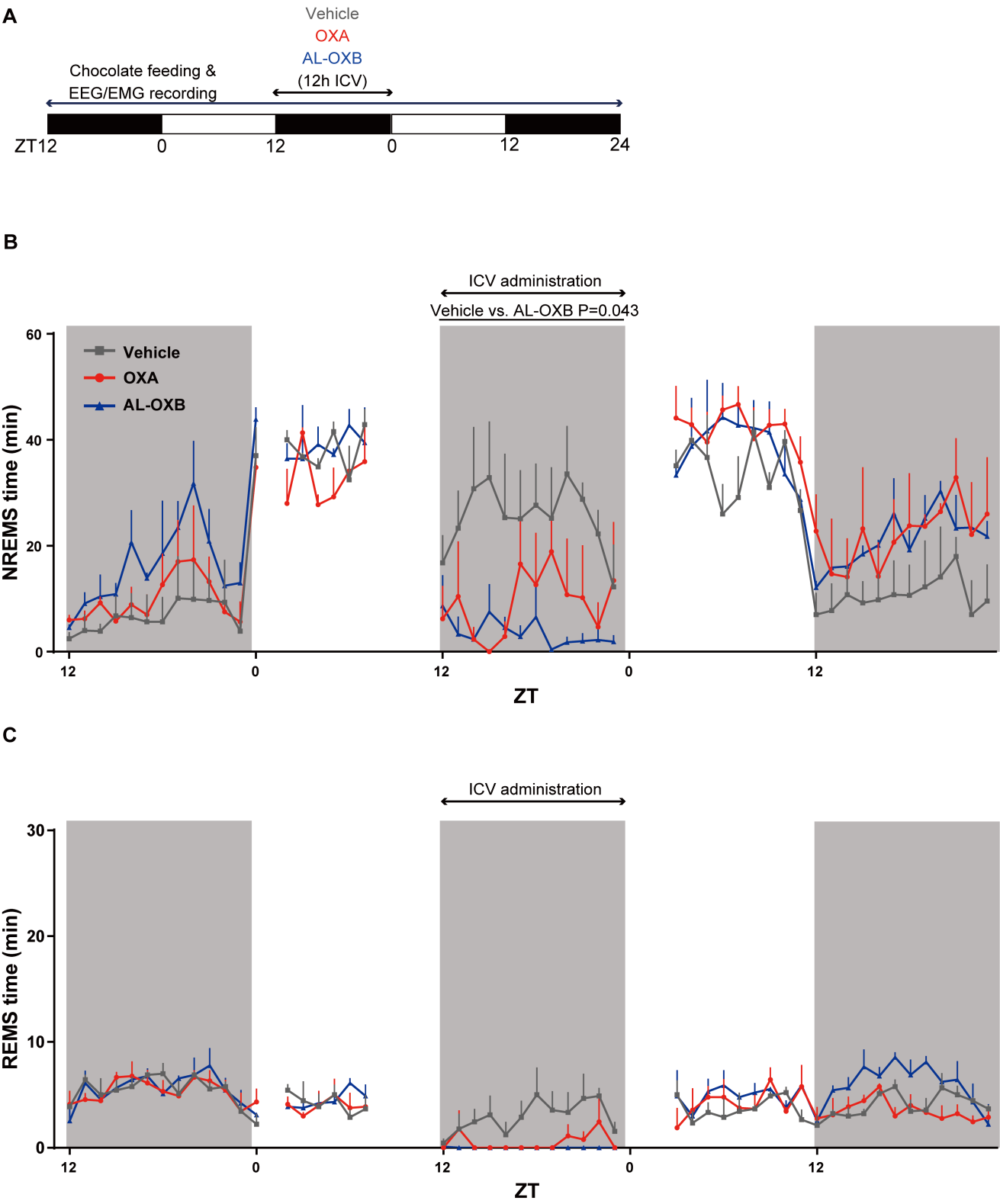

Supplement: S2 Fig — (A) Experimental schedule of ICV infusion and EEG/EMG recording. (B and C) Hourly plots of NREM sleep time (B) and REM sleep time (C) before, during and after continuous ICV administration. Data represent the means ± SEM from 3 mice. Statistical analysis: two-way repeated-measures ANOVA. (PDF) [file pone.0271901.s002.pdf]
