# Supplementary material for: OX2R-selective orexin agonism is sufficient to ameliorate cataplexy and sleep/wake fragmentation without inducing drug-seeking behavior in mouse model of narcolepsy
Source: PLoS One. 2022 Jul 22;17(7):e0271901. doi: 10.1371/journal.pone.0271901 (PMC9307173; doi:10.1371/journal.pone.0271901)
Supplement: Attachment — Submitted filename: Response to Reviewers_final_MY2.docx [file pone.0271901.s004.docx]

**Point-by-Point Responses to Reviewers’ Comments**

Manuscript #: PONE-D-22-06089R1

OX2R-selective orexin agonism is sufficient to ameliorate cataplexy and sleep/wake fragmentation without inducing drug-seeking behavior in mouse model of narcolepsy

We thank the Editor and Reviewers for their highly valuable and constructive criticisms; we believe that we have responded appropriately to all of their comments, which we feel improved the manuscript significantly. In the following sections, the original comments raised by the Editor/Reviewers are indicated in *italic*, and our responses in blue.

**Additional Editor Comments:**

*For clarity and ease of consultation, please report in the manuscript the administered concentrations of AL-OXB and OXA in terms of net peptide. Please indicate the purity and % net peptide content for each peptide.*

We have added requested information in the Reagents section under Materials and Methods (Line 114-118).

*Please clarify whether the data fulfilled the requirements for parametric statistical analysis with ANOVA.*

We have added the description for test of normality in the Statistics section (Line 256).

*Please note that a working URL address to all data underlying the findings or inclusion of the data as supplementary material will be needed before the eventual acceptance of the manuscript for publication.*

We included Excel file containing all raw data in Supporting Information (Line 680).

**Reviewer #1:**

*This is a very nicely done paper describing the possible roles of the two orexin receptors in reversing the symptoms of narcolepsy. The combination of in vitro and in vivo studies is a great strength of the study, as is the conditioned place preference tests. The paper shows the way to a possible treatment of human narcolepsy.

A limitation of the study is the use of orexin KO mice. Although these mice, developed by some of these investigators, led rather directly to the cause of human narcolepsy, the consensus in the field is that human narcolepsy is due to the loss of orexin neurons, not just the orexin peptide. Orexin neurons contain glutamate, dynorphin and Narp. These transmitters are present in the KO mice, but not in human narcoleptics, so the KO mouse results may not exactly align with the pathology of human narcolepsy. The use of the orexin neuron deletion mice developed by Yamanaka might more closely parallel the human condition and comparison of the results of these two models might shed light on the role of the other transmitters in orexin neurons.*

We have added our rationale and justification for using orexin KO mice as a narcolepsy model in the study limitation paragraph under Discussion (Line 468-476).

*In the introduction, I would suggest moving or deleting "and dreaming." Human cataplexy is generally not accompanied by dreaming unless it progresses into a long REM sleep episode. You may want to position these words after "hallucinations." Histaminergics is misspelled.*

We have rearranged the sentence, so that it is less confusing (Line 52-55).

*A very minor point is that the support and animal study questions ask by PLOS One are not properly addressed in their forms, although all the information is in the manuscript.*

We have added the required information such as permit number for the animal protocols (Line 105-106).

**Reviewer #2:**

*The authors examined whether orexin 2 receptor selective agonist (AL-OXB) could effectively reduce cataplexy and sleep/wake fragmentation in orexin knockout mice and got affirmative results. In addition, using wild type mice, the authors showed that ICV administration of OXA but not AL-OXB induced conditioned preference indicating less addiction-inducing effect in the latter. The theme of the study is worth examining and overall conclusion seems convincing. However, there are several points to be clarified.
1) Please report the amount of chocolate consumed in the cataplexy experiment (Fig. 2) because orexin is known to induce food seeking. Was there any possibility that possible change in appetite affected the wake/sleep structure?*

The amount of chocolate consumed (which did not differ among the experimental groups) has been added in the cataplexy experiment paragraph under Results (Line 311-313).

*2) Please specify when of the day pre- and post-test was performed in CPP testing. According to Fig. 5A, it appeared to be during the daytime around ZT3. If it is correct, then mice should be in the resting period and sometimes fall into asleep. Such behavior may distort the preference score. To exclude such possibility, information should be provided about mobility such as the crossing times between the components and/or distance traveled during the observation period.*

We have now added the statement that CPP tests were conducted at ZT4-7 in the relevant section under Results (Line 382); we apologize that this information was somewhat buried in the ICV administration section under Materials and Methods in the original manuscript. Although we did not explicitly measure the indices of mice’s mobility, during the short 15-min periods of pre-tests and post-tests, all mice were quite active and never slept, simply because they were in a new environment. We have added the statement that mice never slept during the 15-min test periods (Line 387-388).

3) In related to the point #2, why the authors selected to test and inject drugs during the resting period whereas the drugs were injected just before the active (night) period in the sleep study? To examine possible side effect (addiction-inducing effect in this case) of a drug, we should use the same dosing schedule in the main effect (sleep study in this case). Don’t you agree?

We have added our rationale for why we did CPP tests during the light phase (resting period for mice) in the relevant paragraph under Results (Line 382-386). However, we do agree with the Reviewer that, in general, it would be more appropriate to perform the tests for the possible side effects at the same time of the day when the drug would be administered for therapeutic effects. Such tests would be highly important in clinical trials in humans. In the present study, we prioritized the sensitivity of CPP tests in detecting the drug seeking behavior.

**Reviewer #3:**

*The manuscript aims at demonstrating that the selective activation of the orexin receptor 2 is sufficient to ameliorate cataplexy and sleep fragmentation in orexin-deficient (ORX-KO) mice without inducing drug seeking behavior as tested using the place preference test.
The originality of this study is to directly compare OXA and AL-OXB in vivo. The authors show that only TMN neurons are activated by AL-OXB (expressing only ORX receptor 2) while OXA activates both, neurons of the TMN and the LC (expressing only ORX receptor 1) as a validation of the selectivity of the compounds for the orexin receptor 2.
Although the study has limits, it is of interest for the design of future therapeutics.
The manuscript is well written.

• Please add the total number of mice used in the study and for each experiments in the method section.*

The total number of mice in the study has now been reported in the Animals section under Materials and Methods (Line 108-109). The number of mice for each experiment has been stated in the corresponding Figure legends (Line 283, 321, 326, 350, 371, 374, 395-396, 669, 677).

*• Chronic icv injections were performed in only 3 mice. No statistical test can be robust with such small number of individuals. Data should be reported with caution and be mainly descriptive (rather than quantitative). Please mention/discuss it in a “technical limitation” section.*

We agree with the Reviewer, and the requested statement has been added to the study limitations section under Discussion (Line 476-478).

*• Figure 1 shows the dose response curves of intracellular CA2+ induced by OXA and L-OXB in vitro. L-OXB is approximately one log more efficient than OXA. However, drugs are used at the same concentration in vivo. Could the authors comment on it? Please discuss it.*

We have added our comments on the apparent discrepancy between in vitro and in vivo potencies of OXA versus AL-OXB in the third paragraph under Discussion (Line 427-431).

*• Figure S1 show an increase in NREM sleep after OXA treatment compared to vehicle at ZT5-7, that is not seen with L-OXB. It might be an additional positive point for L-OXB versus ORXA, but it is not discussed. Could the authors comment on it?*

We have added a brief statement on this point in the relevant paragraph under Results (Line 337-339). We did not go so far as to say that this may be a positive point for OX2R-selective agonists, since more studies are clearly needed to investigate the possible mechanisms for this difference.

**Reviewer #4:**

*This important paper evaluates the efficacy of orexin A (OXA) and AL-orexin B (AL-OXB) in several in vitro and in vivo assays and concludes that Ox2R activation by AL-OXB is sufficient to prevent cataplexy-like episodes and fragmentation of wakefulness in OXKO mice. The work is well-thought out, carefully executed and, with one exception, the results are properly interpreted. That exception is particularly evident on page 17 when the authors conclude the first paragraph with the statement “In other words, the selective activation of OX2R is sufficient to ameliorate both narcoleptic symptoms.” I understand that the authors would like to make this conclusion but, based on the data presented in Figs. 2C, 2D and Fig. 4, the same statement could be made for OX1R since OXA is at least as effective as AL-OXB in all the sleep/wake bioassays shown. The data presented does not exclude this possibility because an Ox1R-selective compound has not been evaluated. As such, I find that statement as well as the title of the paper to be misleading.*

We agree with the Reviewer that, without testing an OX1R-selective agonist (which is not available at this time as far as we know), or perhaps testing a non-selective agonist in OX2R-deficient mice, we cannot formally exclude the possibility that OX1R-selective agonism may also be sufficient to ameliorate the symptoms. Formally speaking, what we can say in the present study is that OX1R activation is not necessary for ameliorating the symptoms, as far as OX2R is activated. Accordingly, we have added an explicit sentence in the end of the relevant paragraph under Results (Line 345).

*The EEG/EMG recordings are scored in 20-sec epochs which is unusually long for rodent studies in which 10-sec epochs are the norm and many labs even score in 4-sec epochs. Although the choice of epoch duration is somewhat arbitrary, longer epochs provide less resolution of the fine structure of sleep architecture. In fact, it is certainly conceivable that all 3 states could occur in a 20-sec epoch. Consider, for example, the sequence NREM-REM-Wake. Consequently, the authors should describe their rationale for such long epochs.

Related to this issue are the determinations of “mean episode duration” as shown in Figs. 2F, 4D and Fig. S1. In fact, Fig. 3 is entirely leveraged on episode duration measurements as the number of state transitions reported in this figure would certainly be larger if 10-sec epochs were used. Because of the fact that rodent sleep is fragmented throughout the 24-h period rather than consolidated as occurs in (most) humans, many sleep researchers establish rules to determine a bout (or episode) of NREM or REM sleep, such as that 3 consecutive epochs of NREM sleep or 2 consecutive epochs of REM sleep must occur before a bout (or episode) is consider to be NREM or REM sleep. The authors do not stipulate any such criteria, which may be due to their use of the long epochs.*

We again agree with the Reviewer. We used long 20-s epochs in sleep staging in this study, knowing that the absolute value for mean sleep/wake episode durations (or the absolute number of stage transitions) is not so important or meaningful as the relative comparison of mean episode durations under different drug treatment conditions. We have added our rationale and justification for using 20-s epochs in the EEG/EMG analysis section under Materials and Methods (Line 184-195). There we have also described the reason why such additional rules for defining sleep/wake “bouts” are not necessary with the 20-s epoch analyses, by taking liberty of partially using the Reviewer’s comment above.

*Neither OXA and AL-OXB at 1 nmol induce CPP but there’s no data to show whether OXA at 1 nmol could prevent cataplexy-like episodes and fragmentation of wakefulness in OXKO mice. Has OXA been evaluated at that dose or not?*

We have added a statement that we did not evaluate OXA at 1 nmol in the relevant paragraph under Results (Line 311).

*What effect does continuous ICV infusion as shown in Fig. 4 have on cataplexy, if any? It would be interesting to know what effect, if any, continuous ICV infusion of OXA and AL-OXB have on body temperature.*

We have added a sentence describing why we did not assess cataplexy in continuous ICV infusion experiments (Line 364-366). We did not measure body temperature in this study, either, since it would require an additional telemetry sensor, which could be rather tricky to use simultaneously with the EEG/EMG monitoring.

*Supp. Fig. 2 suggests that there is a trend toward a rebound in NREM sleep on both the day and subsequent night after cessation of ICV infusion. Although these trends may not be significant on an hourly basis, are they significant over 6-h or 12-h periods? What about NREM delta power?*

We have added our comments on this point in the same paragraph above (Line 361-364).

*On page 17, the authors conclude the next paragraph with the statement “despite the stress.” What stress do the authors refer to? This is the first use of the word “stress” in the paper.*

We apologize for the confusion; the “stress” phrase was a careless leftover from a previous manuscript editing and has now been deleted (Line 360-361).

*On page 20, the authors state “it is likely that higher agonist potency may be required for ameliorating wake fragmentation than suppressing cataplexy.” This is an interesting point that should be developed further.*

We thank the Reviewer for pointing out this important issue; we have added our full comment in the third paragraph under Discussion (Line 438-444).

*Figure 4: why are the error bars so large during the infusion? Figure 4D suggests that AL-OXB is weaker than OXA.*

These error bars are large because: i) episode (bout) duration is an inherently noisy readout; ii) the corresponding mean values are much larger during the infusion; and iii) the number of animals are small (n = 3). These data did not yield statistically significant differences between OXA and AL-OXB.

*p. 10: “administrated” should be “administered”.
P. 17, last sentence would be better English if the authors changed it to read “These results suggest that the administration of an OX2R-selective agonist in sufficient doses to ameliorate cataplexy and fragmentation of wakefulness does not produce addiction-related behavior.”
p. 21, first line: eliminate “the” after “in”.
The authors are not appropriately listed in Ref 25.*

We have corrected these errors exactly as suggested by the Reviewer.
