# Supplementary material for: OX2R-selective orexin agonism is sufficient to ameliorate cataplexy and sleep/wake fragmentation without inducing drug-seeking behavior in mouse model of narcolepsy
Source: PLoS One. 2022 Jul 22;17(7):e0271901. doi: 10.1371/journal.pone.0271901 (PMC9307173; doi:10.1371/journal.pone.0271901)
Supplement: Attachment — Submitted filename: Response to Reviewers.docx [file pone.0271901.s005.docx]

**Point-by-Point Responses to Reviewers’ Comments**

Manuscript #: PONE-D-22-06089R1

OX2R-selective orexin agonism is sufficient to ameliorate cataplexy and sleep/wake fragmentation without inducing drug-seeking behavior in mouse model of narcolepsy

We thank the Reviewers #1 and #4 for their additional comments; we believe that we have responded appropriately to their remaining criticisms. In the following sections, the original comments raised by the Editor/Reviewers are indicated in *italic*, and our responses in blue.

**Reviewer #1:**

*I don't understand what the authors are saying in the second sentence of the Introduction. I suggest shortening it to: "The abnormal gating of REM sleep-related neurophysiological mechanisms, such as REM atonia contributes to cataplexy (sudden bilateral skeletal muscle weakening triggered by a strong emotion) and sleep paralysis." Dreaming is not confined to REM sleep and is not studied (nor could it be) in this mouse experiment.*

We have modified the sentence exactly as suggested (Line 52-54).

**Reviewer #4:**

*The authors' response re the use of the 20-sec epoch is very misleading and incorrect. For example, in a 20-sec epoch, the following sequence of states commonly occurs: NREM-REM-W. This epoch would be scored as one of the 3 states. The purpose of contiguity rules for shorter 4-sec or 10-sec epochs is to obviate this problem when sleep architecture analyses are reported (e.g., number of bouts and mean bout duration per state). The authors' revised text is thus both facile and misleading and must be corrected before I recommend acceptance.*

In order to further verify that the same conclusions are obtained with sleep/wake scoring in 4-s and 20-s epochs, we re-scored the original EEG/EMG recordings for Fig 2F and S1 Fig in both epoch lengths. Results from the 4-s epoch analysis are shown in newly added S1 Fig E and F, which end up very similar to the 20-s epoch analysis shown in Fig 2F (Line 193-195; 664-665). We deleted the following sentence from Results, since it was no longer valid in the re-analyzed data: “Interestingly, OXA but not AL-OXB increased NREM sleep amounts at ZT5-7, possibly representing a rebound sleep (S1 Fig).” We also modified the relevant part of Methods in order to make the descriptions clearer (Line 186-188).
